# Supplementary material for: Identification and analysis of individuals who deviate from their genetically-predicted phenotype
Source: bioRxiv. 2023 Feb 10:2023.02.10.528019. Preprint. [Version 1] doi: 10.1101/2023.02.10.528019 (PMC9934696; doi:10.1101/2023.02.10.528019)
Supplement: Supplement 2 [file media-2.pdf]

Supplementary Information

Phenotypic criteria for filtering genes catalogued in OMIM and described as causal for syndromes associated with stature

- Short stature must not be attributable merely to failure to thrive and/or specific metabolic disturbances and/or intestinal failure/enteropathy and/or very severe disease (e.g. early lethality or severe neurologic disease
- Short stature must also be either consistent (≤ -2 SD in the great majority of patients with data recorded) or present in multiple families/sibships and accompanied by more severe short stature (-3 SD) or skeletal dysplasia (not simply poor bone quality/fractures) or brachydactyly or shortened digits (not clinodactyly or syndactyly) or disporportionate short stature or limb shortening (not simply absence of specific bones)
- Tall stature must be consistent ($> +2$ SD in the great majority of patients with data recorded) or accompanied by more severe tall stature ($> +3$ SD) or arachnodactyly
- Brachydactyly must be not just fifth finger involvement, and either consistent (present in the great majority of patients) or accompanied by consistent short stature or other skeletal dysplasias involving the spine or long bones
- Skeletal dysplasias must involve the spine or long bones (not just the skull or face) and must be accompanied by short stature or brachydactyly as a significant feature (not just clinodactyly or syndactyly), or limb or digit shortening

Supplementary Tables 1-8 of 10

STable 1. 238 Genes with prior evidence for a causal association with height, filtered on those with evidence of a dominant inheritance relationship

ACAN	CILK1	EZH2	HOXA13	MAGEL2	PDE4D	RPS17	SRCAP
ACTB	COL10A1	FAM111A	HOXD13	MAP2K1	PDGFRB	RPS19	SRY
ACTG1	COL11A1	FAR1	HRAS	MAP2K2	PHEX	RPS24	STAG2
AFF4	COL11A2	FBN1	HSPA9	MAP3K7	PIEZO2	RPS26	STAT5B
AKT2	COL1A1	FBN2	IFITM5	MATN3	PIK3R1	RPS28	TBL1XR1
ALDH18A1	COL1A2	FBXO11	IGF1R	MBD5	POGZ	RPS6KA3	TBX1
ALPL	COL2A1	FBXW11	IGF2	MC4R	POLE	RPS7	TBX3
AMER1	COL9A1	FGFR1	IHH	MECP2	POU1F1	RRAS2	TBX4
ANKRD11	COL9A2	FGFR2	IKBKG	MIR140	PPM1D	RUNX2	TBX5
ANTXR1	COL9A3	FGFR3	JAG1	MMP13	PPP1CB	SEMA3E	TBX6
ARCN1	COMP	FIG4	JAK1	MRAS	PPP2R3C	SGMS2	TCF4
ARHGAP31	CREBBP	FLNA	KCNJ2	MSX2	PPP3CA	SHOC2	TGFB2
ARID1A	CSNK2A1	FLNB	KDM3B	MYCN	PRKAR1A	SHOX	TGFB3
ARID1B	CYP11B1	FN1	KDM6A	NAA10	PTCH1	SHOX	TGFBR1
ARID2	CYP19A1	FZD2	KIF22	NEK1	PTDSS1	SKI	TGFBR2
ATP8B1	DDR2	GDF5	KMT2A	NF1	PTH1R	SLC25A24	THRA
ATR	DNMT3A	GH1	KMT2C	NFIX	PTHLH	SLC2A2	THRB
ATRX	DPF2	GHR	KMT2D	NIPBL	PTPN11	SMAD4	TRPS1
BMP2	DVL1	GHSR	KRAS	NLRP3	PUF60	SMARCA2	TRPV4
BMPR1B	DVL3	GLI2	LBR	NOG	QRICH1	SMARCA4	TTC21B
BRAF	EBF3	GLI3	LHX4	NOTCH1	RAI1	SMARCB1	USP9X
BRCA1	EBP	GMNN	LMBR1	NOTCH2	RBPJ	SMARCE1	WASHC5
BRCA2	EFNB1	GNAS	LMNA	NPR2	RIT1	SMC1A	WDR37
BRPF1	EP300	GRIN2A	LOX	NRAS	RNF113A	SMC3	WNT1
BUB1B	ERCC6	H1-4	LRP4	NSD1	ROR2	SNRPB	WNT5A
CAMK2G	ESR1	HCCS	LRP5	OFD1	RPL11	SON	ZBTB18
CBL	EVC	HDAC6	LTBP3	OTX2	RPL13	SOS2	ZC4H2
CCNQ	EVC2	HDAC8	LZTR1	P4HB	RPL26	SOST	ZNF148
CDKN1C	EXT1	HESX1	MAB21L2	PAX8	RPL35A	SOX11	SOX9
CHD7	EXT2	HMGA2	MAF	PDE3A	RPL5		

Genes with prior evidence for a causal association with height, filtered on those with evidence of a dominant inheritance relationship

STable 2. Number of individuals who can be defined as deviating from their genetically predicted height varies substantially be definition

**Controls**	109,858 (69.1%)	
**Method of Defining Deviator Status**	**Number of Individuals short for their polygenic score (% of population)**	**Number of Individuals tall for their polygenic score (% of population)**
**Mahalanobis** (0.001)	150 (0.094%)	94 (0.059%)
**Mahalanobis** (0.05/n)	10 (0.006%)	0 (0.00%)
**Regression** (2 *s.d.*)	3,693 (2.32%)	3,623 (2.28%)
**Regression** (3 *s.d.*)	415 (0.261%)	287 (0.180%)
**GRS Centiles** (1.5 *IQR*)	693 (0.436%)	523 (0.329 %)
**GRS Centiles** (3 *IQR*)	23 (0.015%)	2 (0.001%)
**GRS Ranks** (0.001)	340 (0.021%)	228 (0.0014%)
**GRS Ranks** (1/10,000)	82 (0.052%)	44 (0.0028%)

Number of individuals who are defined as deviating from their polygenic score based on three methodologies, split by relatively tall and relatively short. n = 158,951; IQR = Inter-Quartile Range.

STable 3. % Overlap of individuals defined to be shorter than expected varies substantially between methods

	**Mahalanobis (0.001)**	**Mahalanobis (0.05/n)**	**Regression (2 s.d.)**	**Regression (3 s.d.)**	**GRS Ranks (0.001)**	**GRS Ranks (1/10000)**
**Regression (2 s.d.)**	4.06	0.271	-	-	0.0915	0.0219
**Regression (3 s.d.)**	31.4	2.40	-	-	0.781	0.195
**GRS Centiles (1.5 IQR)**	17.7	1.44	18.8	50.3	0.486	0.117
**GRS Centiles (3 IQR)**	15.3	37.5	0.623	5.54	0.206	0.981
**Mahalanobis (0.001)**	-	-	-	-	0.369	0.468
**Mahalanobis (0.05/n)**	-	-	-	-	0.0264	0.108

% of overlap between the methods used to determine shorter than expected deviators

STable 4. % Overlap of individuals defined to be taller than expected varies substantially between methods. No individuals were classified as being relatively tall when using a Mahalanbobis-based P-value threshold = 0.05/n

	Mahalanobis (0.001)	Regression (2 s.d.)	Regression (3 s.d.)	GRS Ranks (0.001)	GRS Ranks (1/10000)
**Regression (2 s.d.)**	2.59	-	-	0.0629	0.0121
**Regression (3 s.d.)**	21.7	-	-	0.746	0.153
**GRS Centiles (1.5 IQR)**	14.3	14.4	51.1	0.436	0.0841
**GRS Centiles (3 IQR)**	2.13	0.055	0.697	0.0952	0.704
**Mahalanobis (0.001)**	-	-	-	0.258	0.353

% of overlap between the methods used to determine taller than expected deviators

STable 5. Evidence of enrichment remains consistent across different definitions of misalignment amongst individuals who were short relative to their genetically predicted height.

	Mahalanobis (0.001)	Mahalanobis (0.05/n)	Regression (2 s.d.)	Regression (3 s.d.)	GRS Centiles (1.5 IQR)	GRS Centiles (3 IQR)	GRS Ranks (0.001)	GRS Ranks (1/10000)
**LoF 0.001 Variants**	0.014	1.00	1.00×10^{-4}	$1.00{\times}10^{-4}$	$1.00{\times}10^{-4}$	0.025	$9.00{\times}10^{-4}$	0.285
**LoF 0.001 Variants (SS)**	$1.00{\times}10^{-4}$	1.00	$1.00{\times}10^{-4}$	$1.00{\times}10^{-4}$	$1.00{\times}10^{-4}$	$1.00{\times}10^{-4}$	$1{\times}10^{-4}$	1×10^{-4}
**Missense 0.001 Variants**	0.159	0.750	0.377	0.326	0.269	0.623	0.485	0.936
**Missense 0.001 Variants (SS)**	$1.84{\times}10^{-2}$	$3.43{\times}10^{-2}$	$1.00{\times}10^{-4}$	$5.00{\times}10^{-4}$	$1.00{\times}10^{-4}$	$9.58{\times}10^{-2}$	$4.45{\times}10^{-3}$	$4.45{\times}10^{-3}$
**ICD9/10 and GP**	$1.00{\times}10^{-4}$	$1.00{\times}10^{-4}$	$1.99{\times}10^{-4}$	$9.00{\times}10^{-4}$	$3.00{\times}10^{-4}$	$1.00{\times}10^{-4}$	$2.00{\times}10^{-4}$	1.00×10^{-4}
**Short (10)**	$1.00{\times}10^{-4}$	$2.94{\times}10^{-3}$	$1.00{\times}10^{-4}$	$1.00{\times}10^{-4}$	$1.00{\times}10^{-4}$	$1.00{\times}10^{-4}$	$1.00{\times}10^{-4}$	$1.00{\times}10^{-4}$
**TDI**	$1.00{\times}10^{-4}$	0.506	$1.00{\times}10^{-4}$	$1.00{\times}10^{-4}$	$1.00{\times}10^{-4}$	$3.00{\times}10^{-3}$	$1.00{\times}10^{-4}$	$1.00{\times}10^{-4}$
**SSHR**	$1.00{\times}10^{-4}$	$1.00{\times}10^{-4}$	$1.00{\times}10^{-4}$	$1.00{\times}10^{-4}$	$1.00{\times}10^{-4}$	$3.00{\times}10^{-3}$	$1.00{\times}10^{-4}$	$8.70{\times}10^{-3}$

Empirical P-values for enrichment in individuals who are short relative to their genetically predicted height across all deviator definitions. SS = Short Stature Specific; LoF = Loss of Function; SSHR = Sitting Standing Height Ratio

STable 6. Evidence of enrichment remains consistent across different definitions of misalignment amongst individuals who were tall relative to their genetically predicted height. No individuals were classified as being relatively tall when using a Mahalanbobis-based P-value threshold = 0.05/n.

	**Mahal anobis (0.001)**	**Regression (2 s.d.)**	**Regression (3 s.d.)**	**GRS Centiles (1.5 IQR)**	**GRS Centiles (3 IQR)**	**GRS Ranks (0.001)**	**GRS Ranks (1/10000)**
**LoF 0.001 Variants**	0.152	0.763	0.017	0.471	1	0.0434	0.182
**LoF 0.001 Variants (TS)**	0.0237	5.57×10^{-4}	0.0705	3.87×10^{-4}	1.00	1.00	1.00
**Missense 0.001 Variants**	0.327	0.401	0.462	0.235	0.816	0.679	0.795
**Missense 0.001 Variants (TS)**	1.00	0.427	0.425	0.457	0.623	0.766	1.00
**ICD9/10 and GP**	0.477	0.815	0.132	0.135	1.00	0.248	0.818
**Tall (10)**	1.00×10^{-4}	1.00×10^{-4}	1.00×10^{-3}	1.00×10^{-4}	0.382	1.00×10^{-4}	1.00×10^{-4}
**TDI**	0.394	0.127	0.786	0.644	1.00	0.654	0.927
**SSHR**	1.00×10^{-4}	1.00×10^{-4}	1.00×10^{-4}	1.00×10^{-4}	1.00×10^{-4}	1.00×10^{-4}	2.00×10^{-4}

Empirical P-values for enrichment in individuals who are tall relative to their genetically predicted height across all deviator definitions. TS = Tall Stature Specific; TDI = Townsend Deprivation Index; SSHR = Sitting Standing Height Ratio

STable 7. Number of individuals who can be defined as deviating from their genetically predicted LDL-C varies substantially by definition

**Controls**	109,858 (69.1%)	
**Method of Defining Deviator Status**	**Number of Individuals short for their polygenic score (% of population)**	**Number of Individuals tall for their polygenic score (% of population)**
**Mahalanobis** (0.001)	68 (0.05%)	90 (0.067%)
**Mahalanobis** ($0.05/n$)	1 (0.0007%)	2 (0.002%)
**Regression** (2 *s.d.*)	3,085 (2.28%)	3,128 (2.31%)
**Regression** (3 *s.d.*)	287 (0.213%)	290 (0.215%)
**GRS Centiles** (1.5 IQR)	3,191 (2.36%)	3,211 (2.37 %)
**GRS Centiles** (3 IQR)	13 (0.0096%)	15 (0.011%)
**GRS Ranks** (0.001)	215 (0.16%)	222 (0.17%)
**GRS Ranks** ($1/10,000$)	28 (0.207%)	31 (0.023%)

Number of individuals who are defined as deviating from their polygenic score based on three methodologies, split by relatively tall and relatively short. n = 158,951; IQR = Inter-Quartile Range.

STable 8. Q-risk factor definitions

Q-Risk Factor	UKB Data Field
**Age**	21022
**Townsend Deprivation Index**	21022
**Triglycerides**	30870
**High Density Lipoprotein (HDL)**	30760
**Systolic Blood Pressure (SBP)**	4080
**Diastolic Blood Pressure (DBP)**	4079
**Body Mass Index (BMI**	21001
**Height**	50
**Weight**	21002
**Cigarettes Per Day (Cigs Per Day)**	2887
**Alcohol Intake Frequency (Alcohol Freq)**	1558

UKB Data Fields used to derived Q-risk measures
